# The impact of diagnosis-intervention packet (DIP) payment on cost structure of inpatient care—Evidence from a tertiary hospital in China

**DOI:** 10.1371/journal.pone.0336584

**Published:** 2025-11-14

**Authors:** Chenxuan Zhu, Zimeng Li, Fanyu Lin, Lijuan Huang, Keyao Cao, Yuxin Xiao, Jianchen Yang, Juan Zhu, Haoye Li, Wei Li

**Affiliations:** 1 School of International Pharmaceutical Business, China Pharmaceutical University, Nanjing, Jiangsu, China; 2 Department of Pharmacy Administration, School of Pharmacy, Xi’an Jiaotong University, Xi’an, Shaanxi, China; 3 Anqing Municipal Hospital, Anqing, Anhui, China; 4 Department of Biomedical Engineering, The University of Texas at Austin, Austin, Texas, United States of America; University of Lisbon, Institute of Social and Political Sciences, PORTUGAL

## Abstract

**Background:**

Since 2020, China has implemented DIP (Diagnosis-Intervention Packet) payment reform to control medical costs and reduce patient financial burden. The reform was piloted in a representative tertiary public hospital within a provincial DIP pilot city.

**Methods:**

The study used hospital settlement and medication records from January 2019 to June 2023. Interrupted time series analysis (ITSA) and structural change degree (SCD) were applied to evaluate the impact of DIP reform on weekly per capita costs and cost structures for surgical and non-surgical groups. Synergistic effects of health system reforms were also assessed.

**Results:**

From January 2019 to June 2023, total costs for surgical and non-surgical groups decreased by 3.42% and 1.25%, respectively. Drug and surgical costs declined significantly (p < 0.05) in both groups, while consumable costs increased significantly (p < 0.05). The growth rate of total costs slowed (surgical group: β_3_ = −14.10; non-surgical group: β_3_ = −10.76). Total costs in the non-surgical group showed a decreasing trend post-DIP intervention (β_1_ + β_3_ = −3.12). Drug costs (surgical group: β_3_ = −5.50; non-surgical group: β_3_ = −4.11) and inspection costs (surgical group: β_3_ = −3.57; non-surgical group: β_3_ = −1.73) decreased in both groups. Structural change analysis showed a degree of structural variation (DSV) of 10.34% for the surgical group and 5.60% for the non-surgical group. Contribution rates of structural variation (CSV) indicated significant contributions from consumable costs (CSV = 55.83%) and drug costs (CSV = 36.02%) in the surgical group, and inspection costs (CSV = 48.75%) in the non-surgical group.

**Conclusion:**

DIP payment reform led to positive outcomes in the cost structure of inpatient care. However, increases in inspection costs and differences in cost structures between groups need further attention. Future efforts should focus on more precise cost management.

## Introduction

As China’s economy continues to grow, the demand for health care is rising, leading to a steady increase in medical costs [1]. From 2010 to 2020, the total health expenditure in China witnessed a year-on-year growth trend, rising from 1,998.03 billion RMB to 7,217.50 billion RMB, which represents an average annual growth rate of 13.70% [2]. The proportion of total health expenditure in GDP also rose from 4.85% to 7.12% over the same period. In terms of the cost of inpatient care, the average cost per patient in public hospitals increased from 8,833.0 RMB in 2015–11,673.7 RMB in 2021, with an average annual growth rate of 5.76% [3]. The high medical cost has placed burden on patients and made it more difficult for national public healthcare system to allocate resources. With the intention of reducing the burden of medical care and improving the efficiency of health resource utilization, China has engaged in a range of approaches in curbing inpatient medical cost, of which healthcare payment method reform has significantly altered the logic of treatment and behaviors of hospitals from the settlement side, thereby having a substantial impact on the inpatient medical cost [4].

**Fig 1:**
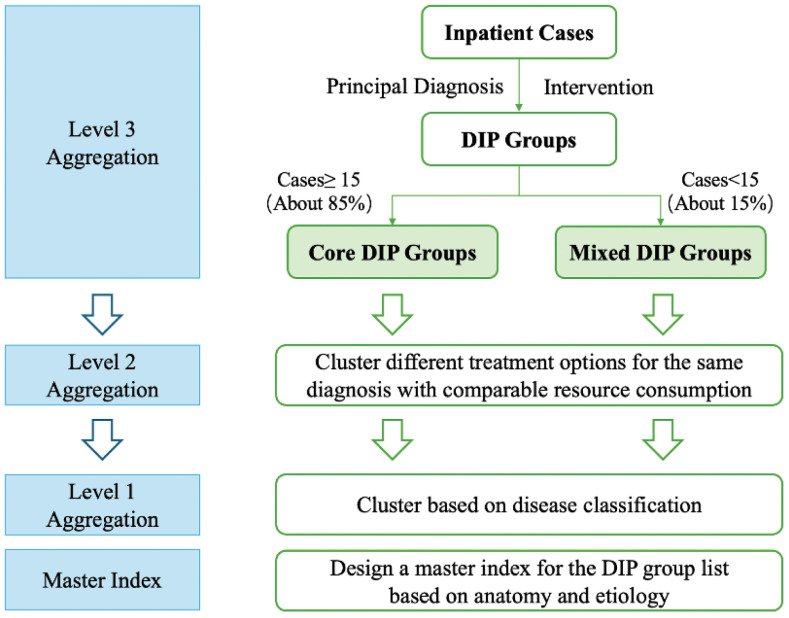
DIP grouping and aggregation system.

Diagnosis-Intervention Packet (DIP) based on big data is an original patient classification and payment system developed in China. This payment system combines the common features of the principal diagnosis (identified by ICD-10 codes) and procedures (identified by ICD-9-CM3 codes) [5]. Based on the common features, medical records are grouped by matching diagnosis and its corresponding interventions and then aggregated three times on the basis of the number of cases, treatment option and disease classification respectively [6] (Fig 1). Specifically, in the first aggregation, those combinations of diagnosis and interventions that contain greater than or equal to 15 cases are clustered into core DIP groups, the others are clustered into mixed DIP groups [7]. The result of classification is published as National DIP Group List. Each prefecture-level city conducts calculations based on local case data and makes fine-tuning adjustments to the National DIP Group List, thereby generating multiple local DIP lists that remain comparable across regions. In brief, such grouping and aggregation approach can objectively reflect the severity of the diseases, the complexity of treatment, the level of resource consumption and clinical behavior norms [8].

**Fig 2:**
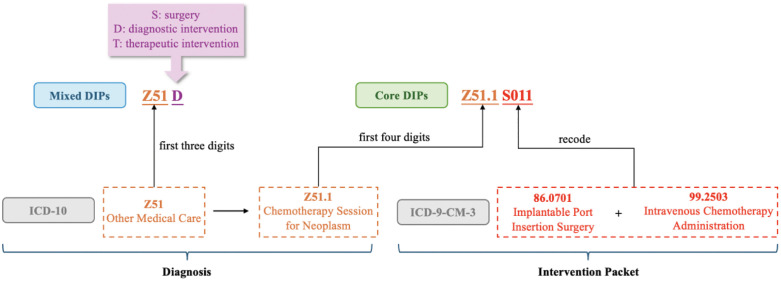
DIP coding logic example.

The groups in the National DIP Group List are coded by at least four digits or letters. In terms of core DIP groups, the first four digits are derived from the corresponding ICD-10 code, representing the principal diagnosis. Starting from the fifth digit, it represents one or more combinations of interventions which are recoded based on ICD-9-CM3. In terms of the mixed DIP groups, codes are fixed at four digits, where the first three digits are derived from the corresponding ICD-10 code, and the fourth digit applies a similar aggregation method to ADRG for treatment categories. There are three situations: (1) D represents diagnostic procedures; (2) S represents surgeries; (3) T represents therapeutic procedures [9]. The coding logic for diseases related to medical care “Z51” is exhibited (Fig 2). It is worth noting that DIP coding is conducted on a city-by-city basis, and there may be differences in coding rules among different cities in real-world application.

The payment standard for each DIP group is determined by the product of its point and point value. Point is a standardized unit for different groups, calculated based on the historical inpatient cost data from the previous three years, weighted in a set ratio. The more severe the conditions and the more advanced the technology used, the higher the point. Point value is the ratio of actual inpatient cost and total points for the year. Therefore, DIP payment is a retrospective healthcare insurance payment method where point value cannot be determined in advance before the year-end settlement [10]. The calculations are as follows:


$${Payment\;Standard}_{i}={\text{Point}}_{\text{i}}\times \text{Point Value}$$ (1)

Point_i_: points for DIP group i for the current year.

Point Value: actual value for each point at the end-of-year settlement.


$${Point}_{i}=\frac{{\text{m}}_{\text{i}}}{\text{M}}$$ (2)

m_i_: weighted average cost for cases in DIP group i over the past three years

M: weighted average cost for all cases over the past three years


$$Point\;Value=\frac{\text{M}}{\sum \left({\text{Point}}_{\text{i}}\times {\text{N}}_{\text{i}}\right)}$$ (3)

M: total cost for all cases in the current year.

Point_i_: points for DIP group i in the current year.

N_i_: the number of cases for DIP group i in the current year.

**Fig 3:**
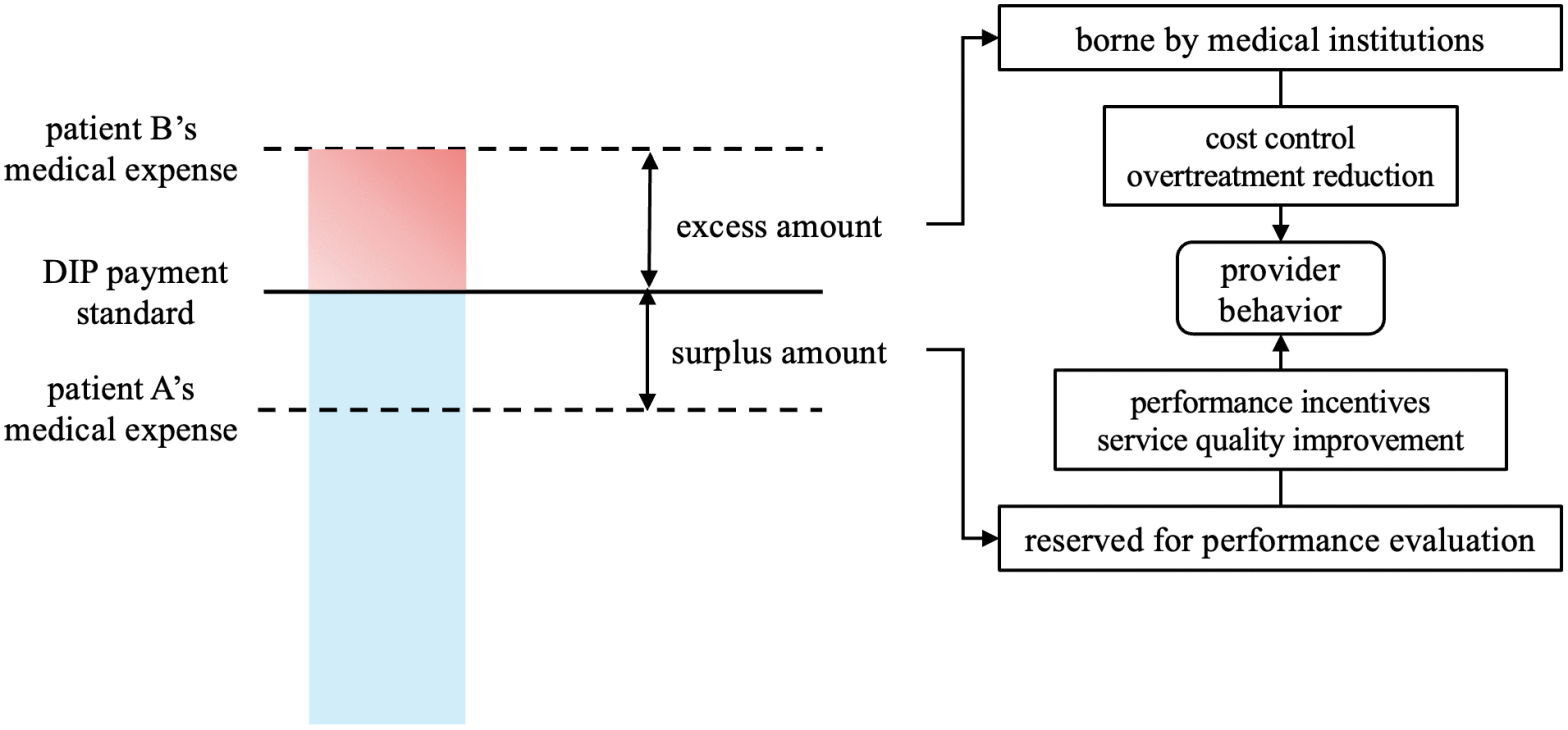
DIP pre-payment and provider behavior mechanism.

Similar to DRG payment, The DIP payment model employs a pre-payment system, through the mechanism of surpluses retained, excesses self-covered, which essentially transforms medical profits into medical costs. This mechanism encourages medical institutions to adopt more cost-effective diagnostic and treatment plans, thereby reducing medical costs [11]. Specifically, when a patient’s actual medical expenses are lower than the DIP payment standard, the medical institution retains the surplus; conversely, if the actual expenses exceed the DIP payment standard, the medical institution bears the excess cost. These surplus or excess amounts are incorporated into performance evaluations. Furthermore, DIP achieves macro-level cost control through regional total amount management. This approach does not directly limit the medical institution’s reimbursement amount but rather controls medical expenses by adjusting the DIP point value. On one hand, overall budgeting effectively reduces the risk of healthcare insurance fund depletion in the region. On the other hand, the more patients a medical institution treats and the greater the difficulty of the cases, the higher the total DIP points, and consequently, the more DIP funds the institution can receive. This approach not only ensures the incentive and constraint effect for individual disease groups but also, to a certain extent, promotes competition among medical institutions in providing medical services, simultaneously controlling medical expenditures and improving the quality of medical services [12,13]. The provider behavior model indicates that hospitals and physicians adjust their service provision based on economic incentives. Particularly, the shift from Fee-For-Service (FFS) to bundled payments under DIP reduces the incentives for the typical moral hazard in healthcare, which is the over-provision of services [14]. Consequently, the DIP mechanism aligns incentives with cost control, encouraging providers to deliver appropriate services within a fixed reimbursement framework (Fig 3).

The aforementioned mechanisms are consistent with cost control theories [15]. Therefore, the key aim is to streamline inpatient medical cost within a normal range by compressing unreasonable cost and optimizing cost structure [5], and to utilize payment reform as a tool to control healthcare spending [16]. Since October 2020, DIP payment pilot programs have been extensively launched in 71 Chinese cities with varying economic levels such as Shanghai, Guangzhou, Luzhou, and Ganzhou. As of July 2024, relatively mature DIP payment has been implemented in over 192 cities in China [17].

Research on the impact of DIP payment on inpatient cost structure has been conducted in academia. For instance, Lai et al. [18] applied the difference-in-difference (DID) method to study the effect of DIP payment on inpatient expenses in major cities. They found a 3.5% decrease in patient expenses after policy intervention, with drug expense contributing significantly to the cost change. Ding et al. [19] analyzed the average inpatient costs and out-of-pocket (OOP) expenses of secondary and tertiary hospitals in DIP pilot cities by interrupted time series analysis (ITSA). The cost in hospitals at all levels significantly declined. Hong et al. [1] conducted the research on the association between medication burden for elderly patients with hypertension and DIP payment, revealing a significant decrease in monthly average medication expenses. Zhao et al. [20] and Wang et al. [21] proved the similar results. Besides, Tang et al. [22] applied structural change degree (SCD) to point out the potential cost transfer after the DRG payment.

However, in the previous research, specific cost components were rarely mentioned. In addition, different trends in medical cost change were witnessed in studies conducted in different periods. Therefore, research about the impact of DIP payment on the inpatient cost structure remains insufficient. Further study is required to explore the similarities and differences in the trends of various cost components, along with their underlying reasons and influencing factors. To fill these gaps, following innovations were introduced in this study. From an academic perspective, firstly, the scope of the study was expanded in respect of the outcome of DIP payment reform. In previous research, indicators of total cost rather than cost structure were primarily analyzed, and we made progress by a detailed analysis of trends of cost components and dig into the changes in cost structures. Secondly, subgroup analysis in consideration of surgeries was made. Patients are categorized into different groups based on whether they undergo surgery, which reflects differences in patient characteristics [23]. The inclusion of surgery during the admission affects the cost structure, as studies have indicated that inpatient costs for non-surgical patients are mainly comprised of drugs cost, while for surgical patients, medical supplies cost is the fundamental part [14]. Therefore, we classified inpatients into surgical and non-surgical groups to compare the differences in cost structure. In addition, this study provides a systematic research framework and methodology for future research on DIP payment reform and other health care payment reforms. Through detailed data analysis and statistical methods, it provides a research path for other researchers to follow. In terms of policy improvement, an in-depth study of the cost structure can provide theoretical support for strengthening cost accounting and refined management. Meanwhile, From the perspective of policy, the collaborative policy effects in China’s health system reform were considered. One notable policy that influences inpatient drug cost is National Volume-Based Procurement (NVBP) initiated in 2019. This policy involves government-led centralized procurement of medical products to substantially cut the prices through manufacturers’ bidding, by which bargaining power can be enhanced through pooling purchasing power across the country [24]. In other words, it launches large-scale procurement in exchange for lower prices and ensures that the purchased medical products achieve a significant level of adoption in hospitals. In addition, due to the sharp price drop of bid-winning brands, manufacturers of bid-non-winning brands are also steered to gradually bring down their prices [25]. As a result, physicians are incentivized to adjust prescription behavior towards varieties covered by NVBP on account of competing prices and performance evaluation [26,27]. Methodologically, previous studies have primarily employed quasi-experimental designs such as DID or ITSA to evaluate healthcare costs and quality. ITSA is suitable for assessing the effects of policy interventions with a clear implementation timeline, as it models changes in level and trend within a single group before and after the intervention. In addition, we applied SCD analysis to examine internal shifts in cost composition, providing more granular insights than trends in overall costs alone [28].

Based on health economics theory, the DIP reform would reduce moral hazard and induce cost-conscious behavior among providers, and works as a long-term intervention, during which both the instantaneous changes and long-term trends in inpatient medical cost can be simultaneously observed [29]. Therefore, we hypothesize that after policy intervention hospitals, in an effort to streamline spending, will strengthen the supervision of healthcare providers’ diagnostic and therapeutic behavior. Consequently, over-treatment will be brought under control, and inpatient costs will drop in the short term. Meanwhile, the trends of cost changes vary among different cost components and subgroups ascribed to factors such as patient characteristics, therapeutic patterns, collaborative policies, etc. However, given the possibility of cost-shifting behavior, inspection costs might not decrease according. Moreover, after initiative-taking budget control, the cost structures of each subgroup will be rationalized. In particular, a decrease in previously redundant cost components may been observed, with additional spending in key clinical pathways, leading to an improvement in medical quality.

Based on the hypotheses, this study aims to collect empirical data from a hospital, analyze the changes among inpatient cost components before and after policy intervention, as well as the reorganization of cost structures, and thus put forward recommendations for refined management of DIP payment reform. A tertiary grade-A hospital was selected for this study, whose all departments implemented DIP payment reform in 2021. This study employed ITSA and SCD to analyze the outcome indicators before and after policy intervention.

## Materials and methods

### Study setting

This study selected a tertiary hospital in Anqing, Anhui Province, as the research sample. In Anqing, data on disease records of hospitals over the past three years were collected, by which a local DIP group database with corresponding point measurement were developed. Besides, DIP system interface renovation and staff training were completed. Starting from February 2021, DIP payment reform was implemented in the hospitals within Anqing, and point values were adjusted annually based on the reform situation.

The sample hospital is the largest hospital in Anqing. As of 2020, the hospital had a complete set of departments and advanced medical equipment which secured nearly 100% bed occupancy rate. In addition, its crucial clinical disciplines accounted for more than 50% of the city’s total number. Therefore, the sample hospital has a higher level of medical expertise and clinical specialty capabilities than other counterparts in this city, which is able to represent the effect of DIP payment reform to a certain extent.

Our research protocol was approved by the Ethics Committee of the sample hospital, which deemed that our study did not involve intervention measures and therefore granted a waiver for informed consent forms. In terms of data access and privacy protection, our data collection took place on May 17, 2024. Throughout the entire data collection process and afterwards, we implemented strict anonymization measures to ensure that all data were stripped of personal identifiers during analysis and reporting.

### Data sources

A total of 461,847 inpatient settlement records of inpatients from 1^st^ January 2019–30^th^ June 2023, which includes 235 weeks, were collected. Patients were excluded from the study if they were uninsured, had insurance but did not receive DIP reimbursement, or had hospital stays that were abnormal or outside the defined study parameters. This resulted in a final study population of 405,389 patients. These records included 12 items which can be categorized by personal information, diagnostic information, and cost information. In addition, medication records during the same period were collected, including 8 items (Table 1).

**Table 1:** Sample hospital settlement and medication record items.

Information type	Information content
personal information	gender, age, healthcare insurance type
diagnostic information	disease type, visit type, department, admission and discharge date
cost information	total cost, consumable cost, test cost, examination cost, drug cost, surgical cost
medication information	generic name, manufacturer, dosage form, specification, dosage, NVBP variety, NVBP batch, bid-winning manufacturer

The missing values were first supplemented by using the difference between total costs and cost components to calculate missing items or using manufacturer and generic name to identify NVBP-related information. Second, data outliers were corrected by merging multiple entries for a single hospitalization for the same patient and deleting illogical data such as negative patient costs and fully duplicated data. The final rate of eliminated data was 1.34%. 401,300 settlement records and 6,051,235 medication records were finally obtained after data cleaning.

### Outcome indicators

DIP payment influences inpatient cost structure through provider behavior change, such as more efficient resource allocation and reduced unnecessary medical services, which in turn lead to stabilization or decline in per capita inpatient costs. In response to the mechanisms, this study designed two patient groups: surgical and non-surgical. The grouping methods, applied based on DIP type and code type, were as follows: first, core DIP groups where surgical procedures were included in intervention packages were categorized into the surgical group, with the rest being categorized into the non-surgical group; second, mixed DIP groups with codes ending in S were categorized into the surgical group, while those ending in D or T categorized as non-surgical.

In addition to grouping, this study set weekly total cost per capita as the indicator, along with its four components: weekly drug cost per capita, weekly consumable costs per capita, weekly inspection cost per capita, and weekly surgical cost per capita. The inspection costs combined both examination and test costs, as there was no significant difference between the two in terms of definitions. Clinically, both were medical services that render assistance to diagnosis, etiology and therapies, only with the distinction in the classification of medical services. Therefore, the combination was reasonable on the basis of practical treatment.

Furthermore, since NVBP policy and DIP payment reform have a synergistic effect on drug cost, and the usage of NVBP varieties is related to physicians’ prescription, the weekly NVBP varieties prescription rate, which refers to the proportion of varieties covered by NVBP among all prescribed varieties, was selected as an extended indicator for drug cost.

### Statistical analysis

#### Descriptive statistics.

We conducted descriptive statistics of the cost data in surgical and non-surgical groups before and after the policy intervention. The Mann-Whitney U test, also referred to as rank-sum test, was employed for hypothesis testing. It assumes that two samples are drawn from populations that are identical except for the overall means, with the intention of determining significant difference in the means of the two independent samples [30].

#### Interrupted time series analysis.

ITSA is a quasi-experimental research design that estimates the effect of an intervention by collecting outcome data at multiple equally spaced time points and comparing the changes before and after the intervention. It is typically assumed that outcome variables before and after the intervention have a linear trend over time, and this analysis can be conducted using a segmented regression model (SRM) for time series data. This study collected inpatient data from 1^st^ January 2019–30^th^ June 2023 at weekly intervals for a total of 235 weeks and set week as time series unit. The sample hospital began to implement DIP payment in February 2021 (week 110). Research suggests that hospitals would gradually adapt to the payment method reforms [19,30], indicating a lag in policy effect. An acceptable lag for healthcare payment reform is 2 months [30]. Therefore, we hypothesized that the first two months (8 weeks) following the policy implementation served as an adaptation period, and thus selected week 118 as the time point for the intervention. We used the model as specified:


$$Y_{t}=\beta_{\text{0}}+\beta_{\text{1}}\times {\text{t}}_{\text{1}}+\beta_{\text{2}}\times {\text{t}}_{\text{2}}+\beta_{\text{3}}\times {\text{t}}_{\text{3}}+\epsilon_{t}$$ (4)

In this model, Y_t_ is the dependent variable, representing the observation at time t. β_0_ denotes the estimated initial level of the indicator. β_1_ denotes the slope before the intervention, indicating the potential trend in the absence of the intervention. β_2_ reflects the level change immediately after the intervention, capturing the instantaneous change at the intervention point. β_3_ represents the change in slope after the intervention compared with that before the intervention, reflecting the sustained effect of policy intervention. The term β_1_ + β_3_ indicates the trend of observed indicator after the intervention, where a negative value signifies a downward trend, while a positive value signifies an upward trend. ε_t_ represents the random error.

To test for potential seasonality in weekly inpatient cost data, we included quarterly dummy variables (Q2, Q3, Q4) in the regression model, using Q1 as the reference category. The results showed that none of the seasonal terms were statistically significant (all p-values > 0.10), indicating that there was no strong seasonal pattern in the data. Moreover, the Durbin-Watson (DW) test is used to check for autocorrelation since the absence of autocorrelation is required in ITSA. A DW value close to 2 indicates no autocorrelation. Our tests revealed that autocorrelation was present in some cost components. Therefore, generalized least squares estimation was used for correction, specifically employing the Prais-Winsten (PW) method to fit the linear regression model. After correction, the issue of autocorrelation in the series was resolved. And Chow test was then conducted, and the results showed that the implementation of the policy led to significant structural changes in all costs (p < 0.001), indicating the existence of the structural break.

#### Structural change degree.

We used the SCD which can reveal the characteristics of changes in the internal composition of medical cost in a comprehensive and objective way. Specifically, results were represented by three indicators: the value of structure variation (VSV), the degree of structure variation (DSV), and the contribution rate of structure variation (CSV) [28]. The calculation was as follows:


$$VSV=x_{i\text{1}}-x_{i\text{0}}$$ (5)

$$DSV=\sum \left|x_{i\text{1}}-x_{i\text{0}}\right|$$ (6)

$$CSV=\frac{\left|x_{i\text{1}}-x_{i\text{0}}\right|}{\sum \left|x_{i\text{1}}-x_{i\text{0}}\right|}\times \text{1}\text{0}\text{0}\%$$ (7)

In this study, i represents the cost indicators. X_i0_ and X_i1_ denotate the initial and final values respectively. VSV is used to assess the magnitude and direction of promotional changes of each cost indicator. A positive VSV indicates that the corresponding proportion has increased, while a negative VSV indicates a decrease. DSV is used to evaluate the overall changes in medical cost, with a higher DSV indicating a larger degree of change. CSV reflects the impact of proportional change of each cost indicator on the overall structure of medical cost. A higher CSV indicates a greater contribution to the overall structural change.

Stata 18.0 was used for statistical analysis. Hypothesis testing was conducted at a significance level of 0.05.

## Results

### General information of the costs for inpatients

A descriptive analysis was conducted to examine the total costs and cost components for inpatients in both groups before and after the DIP payment intervention point in April 2021 in the sample hospital (Table 2). A decrease of total costs for inpatients was observed in both groups, though this change was not statistically significant (p > 0.05). In the surgical group, there was a significant reduction in drug and surgical costs (p < 0.05), while inspection costs showed a marked increase (p < 0.05). In the non-surgical group, drug costs significantly decreased (p < 0.05), but there was a notable rise in both consumable and inspection costs (p < 0.05). Notably, for the whole study period, there was no report of large-scale outbreak of Covid 19 which may influence hospital visit in Anqing.

**Table 2:** Changes in weekly total costs and cost components per capita in surgical and non-surgical groups before and after the DIP payment intervention.

	Surgical group			Non-surgical group		
	Pre intervention	Post intervention	p value	Pre intervention	Post intervention	p value
Total cost	17697.44	17092.64	0.9312	7572.19	7477.40	0.1653
Drug cost	3261.70	3134.85	0.0000	2319.43	2024.26	0.0000
Consumable cost	5556.15	5161.62	0.4114	236.94	241.25	0.0000
Inspection cost	3294.75	3354.27	0.0014	3306.93	3650.23	0.0000
Surgical cost	1925.13	1811.89	0.0000	–	–	–

### Analysis of the cost structure for inpatients in the surgical group

The regression results indicated that both the total cost and cost components for inpatients in the surgical group experienced a significant increase (β_1 _> 0; p < 0.05) prior to policy intervention yet witnessed a significantly instantaneous decrease (β_2_ < 0; p < 0.05) at the intervention point (Table 3).

**Table 3:** ITSA regression results of weekly total cost and cost components per capita for inpatients in the surgical group before and after DIP payment intervention.

	Total cost	Consumable cost	Drug cost	Inspection cost	Surgical cost
Baseline trend (β_1_)	23.79** (11.46, 36.11)	8.76** (4.34, 13.19)	4.65* (1.05, 8.25)	6.36** (4.34, 8.39)	1.33** (0.67, 1.99)
Level change (β_2_)	−1958.41** (−3125.46, −791.37)	−736.92** (−1185.92, −287.93)	−588.27** (−922.22, −254.31)	−396.31** (−586.06, −206.57)	−89.00** (−149.63, −28.38)
Trend change (β_3_)	−14.10 (−30.09, 1.89)	−6.22* (−12.31, −0.14)	−5.50* (−10.48, −0.53)	−3.57** (−6.27, −0.88)	2.14** (1.23, 3.04)
Post intervention trend (β_1_ + β_3_)	9.68 (−0.50, 19.86)	2.54 (−1.64, 6.72)	−0.85 (−4.29, 2.58)	2.79** (1.01, 4.57)	3.47** (2.85, 4.09)

Standard errors: * p < 0.05, ** p < 0.01; (95%CI).

After policy intervention, although long-term total cost was not statistically significant (β_1_ + β_3_ = 9.68; p > 0.05), a significant immediate reduction in overall hospitalization costs was observed at the intervention point (β_2_ = −1958.41; p < 0.01). Among the cost components, inspection cost (β_1_ + β_3_ = 2.79) and surgical cost (β_1_ + β_3_ = 3.47) showed a significant long-term increase (p < 0.05). Yet the rate of increase in surgical cost was higher than pre-intervention (β_3_ = 2.14; p < 0.05). Consumable cost (β_1_ + β_3_ = 2.54) experienced a non-significant long-term change (p > 0.05), while drug cost demonstrated a decreasing trend (β_1_ + β_3_ = −0.85).

**Fig 4:**
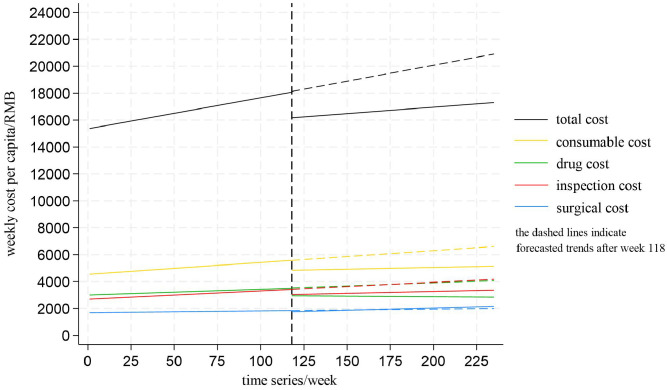
Linear trends of the weekly total cost and cost components per capita for inpatients in the surgical group.

To better illustrate the changes in cost components in the surgical group, linear trends for the whole study period were shown (Fig 4). Forecasted trends after the policy intervention was indicated by a dashed line.

The SCD results (Table 4) showed that the DSV for inpatients in the surgical group was 10.34% throughout the entire study period, reflecting an overall increase of 10.34% in total cost. Among the cost components, consumable cost (VSV = 5.77%), surgical cost (VSV = 0.48%), and inspection cost (VSV = 0.36%) showed positive variations, while drug costs exhibited a negative variation (VSV = −3.72%). This indicated that the proportion of drug cost gradually decreased, whereas the proportions of consumable costs, surgical costs, and inspection costs increased. In terms of absolute value, consumable costs had the highest VSV, indicating that the variation in consumable cost was the most significant after policy intervention.

**Table 4:** SCD results of the cost structure for inpatients in the surgical group.

	Consumable cost	Drug cost	Surgical cost	Inspection cost	DSV (%)
VSV (%)	5.77	−3.72	0.48	0.36	10.34
CSV (%)	55.83	36.02	4.63	3.51	

Moreover, the CSV for consumable cost and drug cost were 55.83% and 36.02% respectively. In contrast, the CSV for surgical cost and inspection cost were only 4.63% and 3.51%. Therefore, in the surgical group, changes in consumable cost and drug cost made greater contribution to the cost structure for inpatients in the surgical group.

### Analysis of the cost structure for inpatients in the non-surgical group

The regression results (Table 5) indicated that there were significant increases in total cost as well as inspection cost, and drug cost for inpatients in the non-surgical group (β_1 _> 0; p < 0.05). In contrast, consumable cost witnessed a downward trend, though this change was not statistically significant (p > 0.05). At the intervention point, there was an instantaneous decrease in total cost (p < 0.05). Specifically, inspection cost and drug cost experienced an instantaneous decrease (p < 0.05), while consumable costs showed a significant instantaneous increase (β_2 _= 44.15; p < 0.05).

**Table 5:** ITSA regression results of weekly total cost and cost components per capita for inpatients in the non-surgical group before and after DIP payment intervention.

	Total cost	Inspection cost	Drug cost	Consumable cost
Baseline trend (β1)	7.64** (5.15, 10.14)	3.52** (2.41, 4.63)	1.89** (0.74, 3.05)	−0.10 (−0.28, 0.07)
Level change (β2)	−317.42* (5.15, 10.14)	−135.36* (−256.04, −14.67)	−165.17* (−299.49, −30.85)	44.15** (22.99, 65.32)
Trend change (β3)	−10.76** (−14.88, −6.65)	−1.73* (−3.31, −0.15)	−4.11** (−6.22, −2.01)	−1.26** (−1.53, −0.99)
Post intervention trend (β1 + β3)	−3.12 (−6.40, 0.16)	1.79** (0.67, 2.91)	−2.22* (−3.98, −0.46)	−1.36** (−1.56, −1.16)

Standard errors: * p < 0.05, ** p < 0.01; (95%CI).

After policy intervention, total cost showed a significant decline (β_3_ < 0; p < 0.01). Its long-term trend was negative while not statistically significant (β_1_ + β_3_ < 0; p > 0.05). Both drug cost and consumable cost demonstrated a significant long-term downward trend (β_1 _+ β_3 _< 0; p < 0.05). In contrast, inspection cost exhibited a notable long-term increase (β_1 _+ β_3 _= 1.79; p < 0.05), though the rate of this increase significantly decreased (β_3_ = −1.73; p < 0.05).

**Fig 5:**
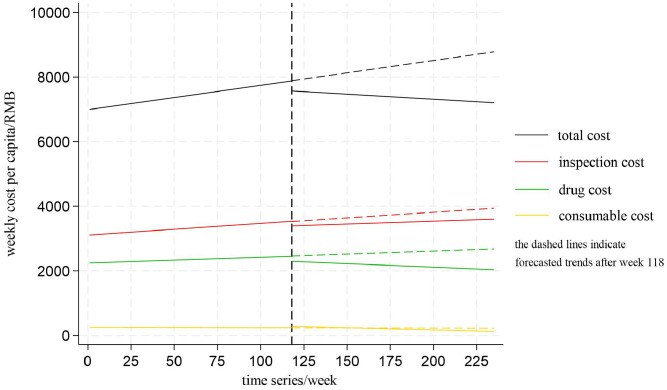
Linear trends of the weekly total cost and cost components per capita for inpatients in the non-surgical group.

To better illustrate the changes in cost components in the non-surgical group, linear trends for the whole study period were shown (Fig 5). Forecasted trends after the policy intervention was indicated by a dashed line.

The SCD results (Table 6) showed that the DSV for inpatients in the non-surgical group was 5.60% throughout the entire study period, which is relatively smaller compared to that of the surgical group. Among the cost components, inspection cost (VSV = 2.73%) showed positive variation, while consumable cost (VSV = −1.57%) and drug cost (VSV = −1.30%) showed negative variation. This indicated that the proportions of consumable cost and drug cost gradually decreased, while the proportion of inspection cost gradually increased. In terms of the absolute value, the VSV for inspection cost was the highest, suggesting that the variation in inspection cost was the most significant after policy intervention.

**Table 6:** SCD results of the cost structure for inpatients in the non-surgical group.

	Inspection cost	Consumable cost	Drug cost	DSV (%)
VSV (%)	2.73	−1.57	−1.30	5.60
CSV (%)	48.75	27.98	23.27	

Furthermore, the CSV of inspection cost ranked the first (CSV = 48.75%), followed by consumable cost (CSV = 27.98%) and drug cost (CSV = 23.27%). Therefore, the change in inspection cost made the greatest contribution to the cost structure for inpatients in the non-surgical group, while changes in consumable cost and drug cost had the similar impact.

### Analysis of prescription rates of varieties covered by National Volume-based Procurement for inpatients

The regression results (Table 7) indicated that distinct trends in prescription rate of varieties covered by NVBP were observed between the surgical and non-surgical groups pre and post policy intervention.

**Table 7:** ITSA regression results of weekly prescription rates of NVBP varieties before and after DIP payment intervention.

	Surgical group (%)	Non-surgical group (%)
Baseline trend (β_1_)	0.0274** (0.0244, 0.0304)	−0.0053 (−0.0094, −0.0012)
Level change (β_2_)	−0.2193 (−0.5970,0.1584)	1.3280** (0.9971,1.6590)
Trend change (β_3_)	0.0029 (−0.0052, 0.0061)	0.0085** (0.0027, 0.0143)
Post intervention trend (β_1_ + β_3_)	0.0025** (0.0230, 0.0326)	0.0021 (0.0008, 0.0074)

Standard errors: * p < 0.05, ** p < 0.01.

In the non-surgical group, the policy intervention led to a significant increase in both the immediate level change (β_2_ = 1.32803%; p < 0.05) and the long-term trend (β_3_ = 0.0085%; p < 0.05) in the NVBP varieties. This suggests that the DIP payment system positively incentivized physicians to prescribe drugs covered by the NVBP.

However, in the surgical group, there were no significant changes before and after the policy intervention (p > 0.05). Nonetheless, there was a higher upward trend compared to the non-surgical group after implementation (β_1 _+ β_3 _= 0.0025%; p < 0.05). This indicates that the DIP payment hardly affected the use of NVBP varieties in the surgical group.

**Fig 6:**
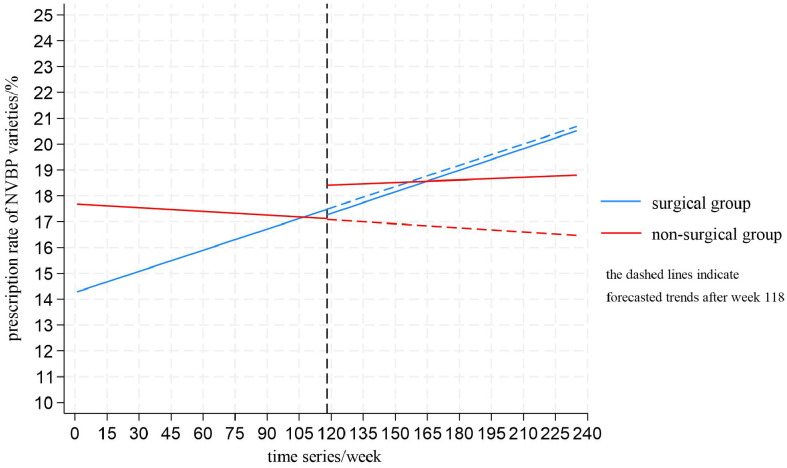
Linear trends of weekly prescription rates of NVBP varieties.

To better illustrate the changes in prescription rate of NVBP varieties, linear trends for the whole study period were shown (Fig 6). Forecasted trends after the policy intervention was indicated by a dashed line.

## Discussion

Our study contributes to the impact of DIP payment reform on medical cost, in particular disentangling different patient groups and cost structures that are widely influenced by DIP payment cost control effects. Overall, DIP payment results in significant short-term transient declines in inpatient medical cost, but the long-term trends are differentiated across patient groups and cost types. Similar findings have been reported for DRG reforms, indicating consistency between the two payment models [14,31,32].

At the initial stages of DIP payment reform intervention, cost management were strengthened by inter-department collaboration [33]. Therefore, the total cost of both surgical and non-surgical group witnessed an instantaneous decrease of 11.71% and 4.27% respectively. However, the changes were not significant before and after policy intervention, which may be attributed to lengthy implementation process of DIP payment reform. Besides, the natural increase in medical cost partially neutralized cost control effect. The total medical cost finally showed a smooth change.

From a long-term perspective, no significant change was witnessed in the total costs for the surgical group. In contrast, the non-surgical group experienced a long-term decrease in total costs following the policy intervention. This decline is due to the fact that before the intervention, there was significant potential for cost reduction in medication and inspection [34]. Consequently, the DIP payment system in the non-surgical group has not yet reached a stabilization phase and may continue to effectively control costs in the long term.

In summary, the hospital should prioritize cost-control management for the non-surgical group by identifying and addressing unreasonable medical practices through regular supervision. Meanwhile, hospitals should boost performance incentives to control surgical costs. This will motivate medical staff to improve diagnosis and treatment processes, ensuring quality while cutting costs [35]. For surgical patients whose total inpatient costs exceed the set payment standards, hospitals can negotiate with health insurance for fair compensation. Methods like fee-for-service, high-rate cases, or special case negotiation mechanism can be used. This balances the financial pressures of DIP payment reform with the need to invest in advanced surgical technologies. It is crucial to achieving long-term policy goals of moderate cost increases while maintaining effective cost control.

In terms of cost structure, the surgical and non-surgical group exhibit distinct features of costs. The total cost of the surgical group exceeded twice that of the non-surgical group, reflecting a significant disparity, and involved cases where multiple surgeries were performed during a single hospitalization. Specifically, while surgical cost is unique to the surgical group, the CSV of only 4.63% indicates a minimal impact on the overall cost structure. Consequently, the disparities arise from differences in treatment characteristics. For instance, consumable cost played a major role in cost-control for the surgical group but had a minor impact on the non-surgical group. In summary, the surgical group incurs higher resource consumption and involve more complex treatments. However, the logic of core DIP grouping of the current version primarily considers comprehensive resource consumption, focusing only on one single surgical procedure and lacking clear rules. This issue has been addressed in the National DIP Group List 2.0 released in July 2024, which stipulates that both the main surgical procedure and associated procedures should be considered, making the grouping logic clearer, and associated procedures with resource consumption exceeding 10% within the group will then be grouped separately.

In terms of drug costs, ITSA results revealed an upward trend in drug costs prior to the DIP payment reform. Following the policy implementation, there was both immediate and long-term reduction in the two groups. SCD analysis showed that the CSV of drug costs was 36.02% in the surgical group and 23.27% in the non-surgical group, while the VSV was −3.72% and −1.30%, respectively, indicating a mild, though substantial in proportion, overall decline in the drug costs’ contribution in cost structure. This trend also aligns with health sector’s strategy, which uses the drug share ratio as a key indicator for measuring cost structure. According to the Scheme of Deepening Health System Reform of the 13th Five-Year Plan from the State Council, the goal is to reduce the drug share ratio in public hospitals to about 30%. In our study, the drug share ratio decreased from 19.30% to 17.14% in the surgical group and from 31.90% to 30.59% in the non-surgical group, which has met the plan’s requirements, thus weakening the reliance on drug revenue to support hospitals’ profit.

As previously noted, NVBP has also significantly impacted drug costs during the same period as DIP payment. We assessed the collaborative effect of NVBP and DIP payment by launching an additional analysis by prescription rate of NVBP varieties. A higher prescription rate of NVBP varieties indicates a greater proportion of usage as well as a larger reduction in overall drug costs. In the surgical group, DIP payment intervention had rare impact on the prescription rate of NVBP drugs. Conversely, in the non-surgical group, the prescription rate of NVBP varieties increased significantly by 0.13% following the intervention and continued to rise. This suggests that DIP payment has a stronger incentive effect on controlling drug costs in the non-surgical group, which may reflect a greater potential for further cost reduction in this group. This phenomenon is primarily driven by the following mechanisms: on one hand, patients in the non-surgical group are predominantly those with chronic diseases or receiving routine treatments, whose medications are largely covered by the NVBP, enabling physicians to more easily substitute original drugs with bid-winning varieties. In contrast, the surgical group has a lower proportion of drug expenditures, and the medications used are often specialized, such as perioperative anti-infective and anesthetic agents, which have limited substitution options. Furthermore, due to the high safety requirements associated with surgical procedures, doctors tend to be more conservative in medication selection. Consequently, the practical impact of the NVBP policy remains relatively limited in the context of surgical drug use.

In terms of consumable costs, ITSA revealed that the surgical group experienced an immediate decrease of 964.25 RMB in consumable cost after policy intervention. Though a long-term upward trend was witnessed, the growth rate was significantly reduced by 76.84% compared to pre-intervention period. SCD showed that the contribution of consumable costs in the cost structure of surgical group increased significantly, with a CSV of 55.83%, compared to only 27.98% in the non-surgical group. While DIP controls surgical and drug costs effectively, inspection costs may rise due to a substitution effect, which is a common phenomenon under cost-containment policies. Providers may increase the use of diagnostics to ensure quality or compensate revenue loss. This highlights that consumable cost is a major focus for future cost-control in the surgical group. Starting in 2020, NVBP began to cover medical consumables, targeting high-value items to reduce costs effectively. It collaborates with DIP payment in the similar way to drugs. However, as it is still in its early stages, the scope covered by the policy requires further expansion. It fails to manifestly alter the overall situation. Meanwhile, advances in surgical techniques leads to increasing consumable costs, which puts forward higher management challenges for hospitals. Currently, there is no single comprehensive method to control the cost of medical consumables. However, more types of consumables are being included in volume purchasing, and local hospitals are improving cost-effectiveness through purchasing alliances. Additionally, provincial areas like Beijing and Zhejiang are implementing policies such as exclusion payment for new technologies and consumables. These measures ensure that patients receive effective treatments without being limited by payment restrictions. They also encourage healthcare organizations to use innovative products, promoting medical technology development and balancing cost control with medical innovation.

In terms of inspection costs, before policy intervention, inspection cost accounted for 18.13% in the surgical group and 44.90% in the non-surgical group, indicating an obviously higher level in the latter. After policy intervention, ITSA depicted that inspection cost in both groups significantly decreased in the short-term and remains a long-term rise with milder growth rate. However, in both groups, the proportion of inspection cost in cost structure increased. This was particularly evident in the non-surgical group, where the inspection cost’s VSV was 2.73%, and its CSV reached 48.75%. Thus, the non-surgical group is the focus for controlling inspection cost. excessive and redundant inspections have long been an issue in hospitals in China. However, as medical technology continues to advance, inspection equipment has become more precise, sophisticated, and comprehensive, offering a wider range of options. This progression has inevitably led to an increase in associated costs. Therefore, the focus of guiding inspection costs is the rationality of its cost growth. Therefore, first, hospitals need to manage their operations more efficiently and create guidelines for the entire treatment process. Second, they should include reasonable testing in staff performance evaluations and monitor and review these practices regularly. Different hospitals should work together to share and recognize each other’s test results. This can reduce the cost of unnecessary tests and lower the financial burden on patients, which is a key part of improving healthcare reform. From the perspective of policies, in China, a public pricing disclosure system has been implemented, requiring hospitals to disclose the billing standards for all medical services, enabling patients to obtain a clear understanding of expected costs prior to receiving care [35,36]. Technology advances also create digital health tools with artificial intelligence (AI) to control costs. Some hospitals are using Clinical Decision Support Systems (CDSS), Electronic Health Records (EHR), or medical information-sharing platforms [37] to help doctors with diagnosis and treatment by AI and big data, which is worth promoting.

The top-level design of DIP payment reform aims to achieve an outcome of tripartite wins for patients, hospitals and the authority alike, by alleviating the financial burden on patients, reducing unnecessary spending for hospital inpatient care, and enhancing the efficiency of healthcare insurance fund utilization. Our study indicates that DIP payment has been effective in controlling inpatient medical costs, but certain cost components still require further reduction. Additionally, the cost structure of the surgical group varied significantly compared to the non-surgical group. The disparities are caused by factors including modalities of treatment, characteristics of patients, varying baseline levels of cost components before policy intervention, and synergistic policies under China’s health system reform.

In order to further improve the effectiveness of DIP payment reform, we have studied the international medical payment model and found that we can learn from the following experiences. First, in medical service pricing, China’s prices for technical labor services are lower than those in developed countries like the UK and Germany [35]. We need to adjust key items to close this gap. Second, in personnel remuneration reform, countries like the US and UK have diverse performance appraisal systems. They focus on feedback and communication to motivate healthcare providers to improve service quality while controlling costs [38]. These reforms typically operate within healthcare systems with relatively autonomous providers and well-established regulatory frameworks. In Chinese context, the public hospital system is characterized by a larger scale, hierarchical decision-making, and close ties to government policy. For example, while performance incentives can motivate clinicians, excessive focus on measurable metrics may risk gaming behavior or undermine intrinsic motivation for quality care. Therefore, a multi-dimensional performance DIP evaluation system is recommended, which not only considers quantitative indicators but also incorporates qualitative measures, including patient satisfaction, clinical outcomes, and adherence to clinical guidelines. This approach can help balance measurable achievements with intrinsic motivation for high-quality care.

Additionally, the US and UK are exploring value-based payment models, such as pay for performance, shared savings, and shared risks. Pay for performance links provider compensation to the quality and effectiveness of care and the patient experience [39]. These models emphasize value over volume, encouraging providers to deliver more efficient and higher-quality care. The efficiency of healthcare insurance funds can be improved [40]. However, these models often require comprehensive data integration, risk adjustment mechanisms, and a mature system for measuring outcomes and patient satisfaction—factors that may still be developing in China, especially in less economically advanced regions. Therefore, pay for performance can be implemented as an auxiliary for DIP payment in regions with better economic conditions and infrastructure to optimize the precision of cost structure control.

Currently, hospitals and policymakers are working together to refine the payment mechanism and ensure financial sustainability. The government has established systems like special case negotiation mechanism and exclusion payment to ensure effective treatment for patients and to enhance the diagnostic and service capabilities of medical institutions. It has also set different reimbursement rates for hospitals of different levels to guide patients to seek appropriate care and promote a tiered treatment system [41]. Looking ahead, policymakers must guide hospitals to strengthen cost accounting and promote refined management of DIP groups. Besides, cost-control goals must be set considering the differences between surgical and non-surgical procedures. It is also crucial to clarify the relationship between revenue, cost and fund reimbursement, by which to establish a reasonable performance assessment model. Finally, joint efforts must be encouraged to promote DIP payment reform [42].

## Limitations

Most notably, as a single-group interrupted time series analysis, it cannot fully account for potential confounding factors such as concurrent policy reforms (e.g., National Volume-Based Procurement, hospital performance management changes, or adjustments in service pricing), economic fluctuations, or other health system interventions occurring during the study period. Furthermore, regional or institutional factors, such as changes in hospital management, local health insurance policies, or population characteristics, may also influence the observed outcomes. As a result, future studies employing multi-site designs or alternative quasi-experimental approaches are warranted to further validate the findings and strengthen external validity.

## Conclusions

In this study, we analyzed settlement and medication records from inpatients at the sample hospital from January 2019 to June 2023. Taking the delay in policy implementation into consideration, descriptive statistics, ITSA and SCD were applied. We found that the introduction of DIP payment policy to certain degree controlled inpatient medical costs and optimized the cost structure. Specifically, the outcome of the surgical group aligned more closely with policy expectations. Notably, drug costs and their proportion decreased significantly. When examining the combined impact of NVBP and DIP payment, a stronger incentive in lowering drug costs was observed in the non-surgical group. Following the policy intervention, the overall trend in consumable costs was promising, yet in the surgical group it took up a higher proportion, remaining long-term management. The growth rate of inspection costs declined, addressing the long-standing issue of over-treatment. In brief, DIP payment reform has demonstrated preliminary success.

To move one step forward towards DIP payment reform’s top-level design of fulfilling tripartite wins, we recommended the following measures. First, it is essential to dynamically update local core DIP groups concerning surgeries based on the inclusion of associated surgeries in the National DIP Group List, with data being meticulously tracked. Additionally, refined management at the cost structure level is necessary to enhance hospitals’ cost accounting. To be specific, unreasonable and redundant treatments, especially in the non-surgical group must be scissored for better quality of care. The proportions of consumable cost in the surgical group and the inspection cost in the non-surgical group should be supervised to avoid rapid growth. Considering the complexity of these requirements, performance assessments designed at the department level must be enhanced to improve the expertise, discipline and synergy of medical practitioners. By adopting these measures, virtuous collaboration can be fostered among all stakeholders for promoting the fulfillment of DIP payment reform.
